# A COVID-19 call center for healthcare providers: dealing with rapidly evolving health policy guidelines

**DOI:** 10.1186/s13584-020-00433-x

**Published:** 2020-12-02

**Authors:** Aharona Glatman-Freedman, Michal Bromberg, Amit Ram, Miri Lutski, Ravit Bassal, Olga Michailevich, Mor Saban, Dvora Frankental, Rita Dichtiar, Anna Kruglikov-Moldavsky, Violetta Rozani, Dolev Karolinsky, Tali Braun, Inbar Zuker, Lital Keinan-Boker, Barbara G. Silverman

**Affiliations:** 1 The Israel Center for Disease Control, Israel Ministry of Health, Ramat Gan, Israel; 2 Department of Epidemiology and Public Health, School of Public Health, Tel Aviv University School of Medicine, Tel Aviv University, Tel Aviv-Yafo, Israel; 3 School of Public Health, University of Haifa, Haifa, Israel

**Keywords:** COVID-19, Pandemics/prevention and control, Call centers, Public health

## Abstract

**Background:**

Communication between health authorities and healthcare providers is an essential element of the response to public health emergencies. Although call centers can facilitate such communication, no published reports describing their outcomes exist. In advance of the expected COVID-19 outbreak in Israel, the Israel Center for Disease Control established a call center dedicated to queries from healthcare professionals.

**Methods:**

The call center operated from February 5, 2020 (week 6) to May 14, 2020 (week 20). Data on calls received, including date and time, caller characteristics, questions and responses were recorded in a database designed for this purpose. The volume, sources and content of queries were analyzed.

**Results:**

In 15 weeks of operation, the call center responded to 6623 calls. The daily number of calls ranged from 1 to 371 (mean 79.8, median 40), peaking on week 12, 2 weeks prior to a peak in new COVID-19 cases. Callers were predominantly physicians (62.4%), nurses (18.7%) and administrators (4.4%). Most worked in primary care clinics (74.2%) or hospitals (8.7%). Among physicians, 42.3% were family physicians or internists, and 10.0% were pediatricians.

The issues most commonly addressed were home quarantine (21.6%), criteria for suspected cases (20.6%), and SARS-CoV2 testing (14.1%). Twenty-five percent of questions involved requests for clarifications of MOH guidelines regarding travel restrictions, clinic management, triage of symptomatic patients, routine medical and dental care, recommended precautions for health care workers with preexisting medical conditions, and other matters. A total of 119 queries were not resolved on the basis of existing guidelines and were referred to MOH headquarters.

**Conclusions:**

This is the first report of a call center established to serve the needs of healthcare providers seeking guidance on COVID-19 management, and to facilitate communication of providers’ concerns to the central health authority. Our work indicates that a central call center for healthcare providers can facilitate the development, implementation and amendment of guidelines and should be an integral element of the early response to public health emergencies. Real-time analysis of the call data may reveal important trends requiring prompt attention.

**Supplementary Information:**

The online version contains supplementary material available at 10.1186/s13584-020-00433-x.

## Introduction

In December 2019, a cluster of cases of acute respiratory illness associated with a previously unrecognized coronavirus (later known as SARS-CoV-2) was reported in the city of Wuhan, Hubei Province, China [1]. The virus spread rapidly throughout the world [2] and on March 11, 2020, the World Health Organization declared COVID-19, the disease associated with SARS-CoV-2, a pandemic [3]. In response to the expected outbreak of COVID-19 in Israel, the government took multiple actions aimed at reducing the number of cases imported to the country, limiting the local spread of the disease, and establishing sufficient testing and treatment capacity.. The COVID-19 response included development and dissemination of numerous new guidelines and directives addressing a wide range of issues, such as: travel restrictions, situations requiring quarantine, COVID case definition, criteria for PCR testing, use of personal protective equipment (PPE), clinic management, and management of patients in quarantine.

Effective communication between health authorities and healthcare providers is an essential element in the response to a major public health emergency [4]. The establishment of a national call center to provide a reliable source of information for health care providers is a component of the Israel Ministry of Health (MOH) emergency response plan to major disease outbreaks [5]. A call center was previously established in preparation for the pandemic H1N1 influenza in 2009.

In accordance with the emergency response plan and in order to support healthcare personnel dealing with rapidly changing COVID-19 directives and recommendations, the Israel Center of Disease Control (ICDC) was directed by the MOH headquarters (HQ) to establish a dedicated call center to respond to inquiries pertaining to COVID-19-related healthcare issues. The call center operated independently of another MOH call hotline, which responded to inquiries from the general public.

Although national and state health authorities from several countries have publicized COVID-19 resources for health care providers, to date, no published reports describing and analyzing the operations of these services. In this work we describe and analyze the operation of the call center established by the ICDC (which belongs to the Israel MOH) in order to facilitate effective communication with health care providers during the early stages of the COVID-19 public health emergency.

## Methods

### Framework

The call center was established on 5 February 2020 (calendar week 6) and was in operation until 14 May 2020 (calendar week 20). Call center staff were all employees of the ICDC with advanced degrees and training in one or more of the following fields: Medicine, Nursing, Epidemiology, Public Health, Preventive Medicine, and Microbiology. The telephone number for the call center was distributed in MOH announcements, in correspondence with the four Health Maintenance Organizations (HMO) of Israel and with healthcare facilities’ management. In some cases patients were advised by their providers to contact the call center rather than the official MOH hotline. The call center operated from Sunday to Friday, with the exception of national holidays, in parallel with the operations of most physicians’ offices in Israel. Hours of operation from Sunday to Thursday were 8:00 to 20:00 and staffing level varied in response to call volume; on Fridays a single staff member was on call from 08:00 to 13:00. The call center staff included at least one physician at all times, who was available to other staff members for consultation.

### Data recording

A dedicated database platform with an interface for recording the details of each call were developed specifically for the call center, The data collected were date and time of each call, identifying information of the caller, the question posed by the caller, and the response provided. In cases in which a caller had more than one question, details of each one were recorded separately. Calls were managed according to the flow diagram shown in Supplemental Figure S1.

Each question was classified according to its content into one of several categories such as: case definition, quarantine, laboratory tests, proper use of personal protective equipment (PPE) by patients and staff, and others.

Responses were provided in accordance with the most current MOH guidelines and directives, which were frequently amended in response to changes in patterns of COVID-19 incidence overseas and in Israel and the evolving knowledge base. In cases in which the available guidelines did not address a specific need raised by the caller, the issue was directed to the most appropriate department within the MOH for consultation and clarification. Where no adequate response was available based on the guidelines and recommendations, the matter was directed to MOH headquarters (HQ).

### Data retrieval and analysis

The crude number of calls received by the call center was retrieved for each day of the call center’s operation and plotted on a daily time line. The number of new daily COVID-19 cases during the call center operation period was retrieved from publicly available MOH data [6].

The number of calls received by the call center were retrieved by category and aggregated per the entire activity period and by week. The numbers of weekly calls per category were plotted on a weekly timeline.

The queries presented to the MOH headquarters were retrieved by categories and aggregated by week. The numbers of weekly queries per category were plotted on a weekly timeline.

The callers’ profession, work setting and municipality were retrieved from the data base. Municipalities were aggregated into districts and percentages of the different professions, work settings and districts were calculated.

Statistical analyses were conducted in SAS enterprise 7.1 (SAS Institute Inc.).

## Results

### Call volume and pattern

**Fig. 1:**
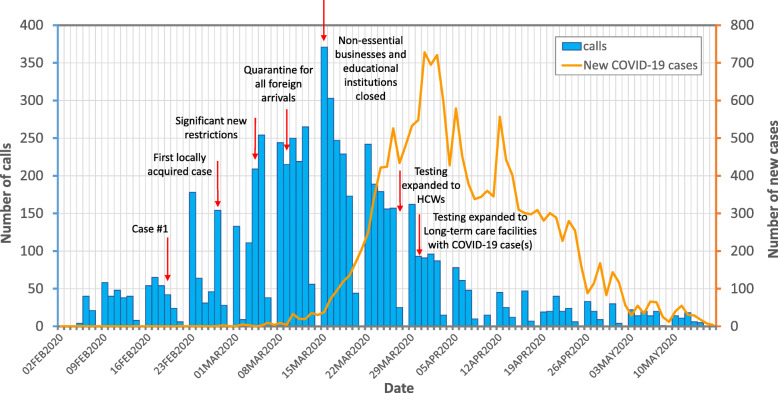
Frequency of calls made to the ICDC call center by date (blue bars), February 5, 2020 to May 14, 2020. Orange curve line represents the number of new COVID-19 cases by date. Red arrows designate relevant key events/guidelines changes

As of 14 May 2020, the call center had received 6623 calls, over 104 days of operation. Frequency of calls by date is shown in Fig. 1. The number of calls per day ranged from 1 to 371 (mean calls per day 79.8, median calls per day 40). The call center became operational 2 weeks prior to the diagnosis of the first case in Israel [5]. The peak period of the call center activity was during the month of March 2020, when mean call volume was 173 per day with the peak number of calls per day (*n* = 371) occurring on week 12, 2 weeks prior to the date on which peak COVID-19 incidence occurred. Call volume was highest on Sundays (mean 118) and lowest on Fridays (mean 20). Increases in call volume that occurred on other week days were associated with distribution of new information such as noteworthy new cases or significant restrictions (Supplemental Table S1).

### Categories of callers

Table 1 presents callers’ characteristics. The most common callers were physicians (62.4%) followed by nurses (18.7%) and administrative personnel (4.4%). Most callers worked in primary care community clinics (74.2%) or hospitals (8.7%). A minority of the callers (< 1%) belonged to emergency services, local departments of health, or mother-child centers. Several callers were private citizens referred to the call center by their healthcare providers. Among physicians, 1750 (42.3%) were family physicians or internists, 412 (10.0%) were pediatricians, 459 (11.1%) belonged to other specialties and for the remainder 1514 (36.6%) specialty was unknown.

**Table 1:** Characteristics of callers to the ICDC call center, February 5 to May 14, 2020 (*N* = 6623)

Callers’ characteristics (***N*** = 6623)		
	N	(%)
**Caller profession**		
Physician	4135	62.4
Nurse	1235	18.7
Emergency service provider	27	0.4
Medic	9	0.1
Administrator / medical secretary	290	4.4
General public	162	2.5
Other	731	11.0
Missing	34	0.5
Total	6623	100
**Work setting**		
Outpatient clinic	4915	74.2
Hospital	575	8.7
Emergency services	29	0.4
Local Department of Health	20	0.3
Mother-child clinic	12	0.2
Other	1071	16.2
Missing	1	0.0
Total	6623	100
**District**		
Northern (16.1% of Israel’s population)	589	13.9
Haifa (11.5% of Israel’s population)	341	8.0
Tel Aviv (15.9% of Israel’s population)	403	9.5
Central (24.5% of Israel’s population)	1245	29.3
Jerusalem (15.9% of Israel’s population)	860	20.2
Southern (14.5% of Israel’s population)	593	14.0
Judea/Samaria (4.8% of Israel’s population)	216	5.1
Total	4247	100

A total of 4568 (69%) of the calls were from callers who identified themselves as employees or contractors of HMOs. All four Israel HMOs were represented by the callers [7].

A total of 4247 (64%) of the calls were from callers who identified the geographic location of their workplace (Table 1). Of those, the highest number of calls, constituting nearly 30% of them, originated from the Central district, which accounts for approximately one-quarter of the Israeli population. The smallest number of calls (5.1%), originated from the Judea/Samaria district, which accounts for 4.8% of the Israeli population.

### Categories of questions

A total of 6933 questions were received during the operation period of the call center. For 143 calls (2.1% of all calls), the details on the specific question posed and the answer provided were unavailable. The questions posed by callers were classified into the categories presented in Supplemental Table S2. The specific issues most commonly addressed were home quarantine (21.6%), criteria for a suspected case (20.6%), and SARS-CoV2 testing (14.1%). Approximately 25% of questions involved requests for clarifications of MOH guidelines regarding issues such as travel restrictions, clinic management, triage of symptomatic patients, providing routine medical and dental care, recommended precautions for health care workers (HCWs) with preexisting medical conditions, and other matters. Examples of the most common question topics for each category are presented in Supplemental Table S3.

**Fig. 2:**
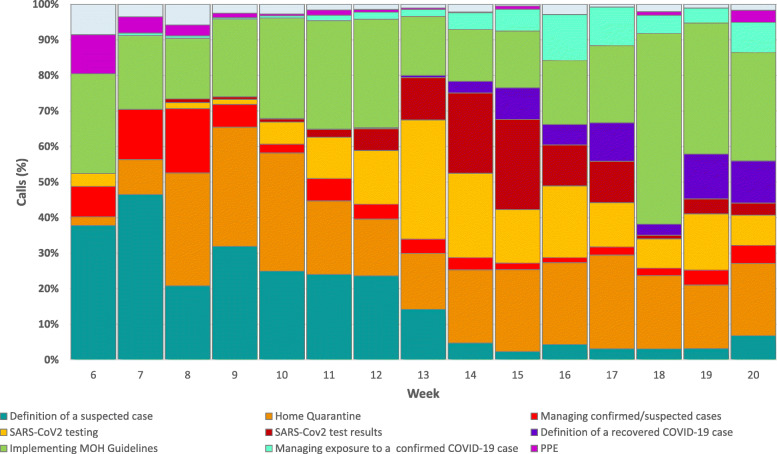
Distribution of questions presented to the ICDC per week, by category

The distribution of questions by category and by calendar week during the call center operation is shown in Fig. 2. Questions regarding home quarantine and implementation of MOH guidelines were addressed throughout the period of operation of the call center. Questions regarding the definition of a suspected case were most commonly addressed during weeks 6 to 12 (14–47% of questions per week), and were less frequent during weeks 14 to 20 (2–7% of questions per week). Questions regarding SARS CoV2 testing and test results were infrequent in weeks 6 to 10 (0–12% of questions per week), and were most frequent during weeks 13–16 (12–24% per week). Questions regarding the definition of a recovered COVID-19 case were not presented by callers prior to week 13.

#### Referrals to MOH headquarters

During the call center operation period, 119 queries were referred to the MOH HQ.

**Fig. 3:**
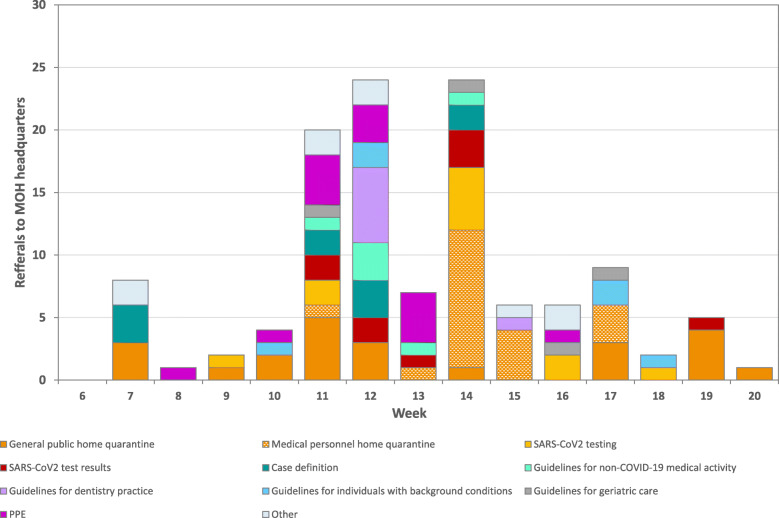
Distribution of referrals made to Ministry of Health headquarters per week, by category

The distribution of referrals by category and by calendar week is shown in Fig. 3.

The largest number of queries was referred on weeks 11, 12 and 14, in a distribution similar to that of the overall volume of calls.

The most common referral category related to home quarantine (36.1%), which in turn could be divided into referrals related to the general public (patients) (19.3% of all referrals) and referrals related to healthcare professionals (16.8% of all referrals). The majority of referrals regarding home quarantine related to the receipt of a text or voice message with instruction to enter home quarantine due to an exposure to a confirmed COVID-19 patient. These messages originated from a dedicated system that was established to locate individuals exposed to COVID-19 patients, based on mobile telephone signals. The referrals mostly pertained either to patients claiming that they were not present at the location/time of the alleged exposure, or to medical personnel who claimed that they were protected by adequate PPE while present on the premises of a medical facility at the time of the alleged exposure. In response to the many calls received regarding this issue, the MOH established a mechanism for appealing an order to enter quarantine, accessible via chat, Whatsapp and the MOH hotline for the general public [8].

The following are examples of changes in MOH policy that grew out of queries to the call center:

1. Publication of detailed guidelines for the operation of dental offices, and the delivery of dental care [9]
2. Specific guidelines for the care of individuals residing in institutions of geriatric care and for their staff. Furthermore, a special program was established to oversee the management of COVID-19 related issues in nursing homes and assisted living facilities (“Fathers and Mothers Shield”) [9]

## Discussion

Although all Israel COVID-19 MOH guidelines were published on a dedicated MOH website, the sheer volume of new information made it more practical for busy providers to contact a single central source of information than to search the MOH website. The call center offered the country’s health providers a centralized source of information tailored to their specific needs, at a time when major and rapid changes in health care delivery were being made.

Our data demonstrates that the call center became operational 2 weeks before the diagnosis of the first COVID-19 case in Israel, and about 8 weeks prior to the first observed peak COVID-19 incidence. These finding indicate that the call center offered the health care community an increased level of support well in advance of the projected need, providing them with the opportunity to become more familiar with the new practices for prevention and treatment.

The decline in call volume prior to the peak incidence of COVID-19 cases reflects the assimilation of MOH guidelines by health professionals, the simplification of guidelines regarding foreign travel (all returnees from foreign travel were required to enter home quarantine, as compared to those arriving from certain countries), and the establishment of COVID-19 call centers and support mechanisms by each HMO [10–13].

The call center question categories indicate the wide scope of questions presented to the call center such as quarantine, SARS-CoV2 testing, use of PPE, as well as management of known or suspected COVID-19 cases and others. The different distribution of the various call categories throughout the call center period of operation reflects the dynamic nature of this public health emergency and the rapid changes in guidelines. The fact that the majority of calls originated from primary care clinics suggests that primary care clinics confronted most interactions with COVID-19-related questions or that guidance and support might have initially been less available for primary care providers in the community than for the staff of inpatient facilities. The fact that only 10% of physicians who called were pediatricians is consistent with the reports that children infected with SARS-CoV-2 have less severe symptoms [14] and lower incidence as compared with adults [15].

The calls originated from all districts of Israel, although the Jerusalem and Central districts were overrepresented relative to their shares of the total Israeli population. The factors that may have contributed to this overrepresentation may be related to the fact that the city of Jerusalem, the most populated city in Israel, had the largest number of COVID-19 cases in Israel, and the Central district has a larger number of physicians practicing family medicine than any other district [16].

In addition to serving as a source of information, an equally important function of the call center was to serve as a channel for raising issues that were not sufficiently clear to healthcare providers or for which guidelines were unavailable and bring them to the attention of the MOH HQ. Since the ICDC is part of the Israel MOH, call center staff were able to bring such issues directly to the attention of the MOH HQ, providing an opportunity for the MOH to amend existing guidelines as well as write new ones in accordance with the needs of the healthcare community.

Effective communication between health authorities and providers in the community is an essential element in the response to a major public health emergency [4]. Interviews with Canadian family physicians who had practiced during public health crises revealed that respondents agreed that a system for disseminating crucial information must be in place before a crisis occurs, and valued having a single source of authoritative information [17]. Respondents also emphasized the need for updates to be concise and targeted to their needs, as physicians rarely have time in the midst of a crisis to review lengthy documents [17].

A variety of methods may be used to facilitate rapid transfer of information on new public health directives and guidelines, each having its advantages and limitations. Text messaging, email, and fax have been found to be effective in the communication of public health messages to health care providers [18], but are practical only for the transmission of relatively short, focused messages rather than for complex and lengthy documents. Furthermore, when updates are published frequently or by multiple sources, the volume of information transmitted may adversely affect recall of the content of messages, or cause providers to ignore or block them [4, 19]. Internet sites featuring a comprehensive listing of relevant documents and “frequently asked questions” are a valuable resource for health care providers and the general public, but must be properly organized to be useful and may not be the best source of quick answers for busy practitioners. These methods of communication, used judiciously, can be useful for rapid dissemination of information to health care providers. None, however, facilitates two-way communication, which is a crucial element of the response to public health emergencies, as providers in the community have an important role in alerting authorities to events of concern and issues that have not yet been adequately addressed [17].

Television and radio also have been used to transmit information during public health emergencies. However, these media are useful for the transmission of short and focused prevention messages to the public [20–22], medical professionals found them inadequate [23].

News conferences were used during the COVID-19 pandemic in Israel to announce changes in public health policy and national response to the pandemic. The messages that were transmitted during these conferences were short, and usually were associated with an increase in the volume of calls received by the call center. This increase in call volume indicated that news conferences alone were insufficient for transmitting information to healthcare professionals.

To date, little data are available regarding the methods used by health authorities to support health care professionals during the COVID-19 pandemic. The World Health Organization lists on its “COVID-19 Health System Monitor” numerous strategies for communicating with the general public, including hotlines, websites and messaging applications but mentions only online information portals as a medium for communicating with health care professionals [24]. A number of national and state health authorities have publicized resources for health care providers. The US Centers for Disease Control and Prevention (CDC), offers a website with a range of resources for providers, schedules periodic ‘Clinical Outreach and Communication’ calls and webinars for provider training, and operates a ‘Clinician On-Call Center’ accessible by telephone to health care providers [25]. The National Health Service in England offers a hot line for providers that can be accessed by telephone or text message [26]. No published reports are available to describe the operations of these or other services directed toward health care providers in the community.

Our work has several limitations. Due to the large volume of calls, caller details could not be completed for some calls. However, because we were able to obtain information for the majority of calls, our analysis does reflect the overall callers’ profile in terms of their parent organization, their profession and their geographic location. Additionally, for 143 callers, no information on the question posed was available. However, since this number represents only 2.2% of the calls, this should have had little or no effect on the analysis of the questions.

## Conclusion

The volume and timing of calls received by the call center support our assumption that it served a temporary essential function, facilitating rapid information transfer to providers in the community at a time when HMOs were putting their own systems in place. Our experience indicates that a central call center for health care providers should continue to be an element of the early MOH response to public health emergencies. In addition, we suggest the following actions to optimize two-way communication between health authorities and providers during a public health emergency:

1) Development of data systems that facilitate real-time analysis of call data, shortening the time required to identify significant trends in the subject of calls, geographic location of callers and other factors of importance.

2) Rapid evaluation, prior to and during the management of a health emergency, of the effectiveness of the services provided by health authorities, in order to determine how best to meet the needs of providers. Brief internet surveys have the potential to gather rapid feedback from providers in the community [27] and allow authorities to identify unmet needs.

3) Expanding the available methods for providers to make contact with the MOH in emergency situations to include email and chat as well a telephone call center.

## Supplementary Information

**Additional file 1: Supplemental Table S1.** Timeline of events and guidelines related to the national response to COVID-19.

**Additional file 2: Supplemental Table S2.** Categories of questions raised by callers to the ICDC call center (*N*=6,933).

**Additional file 3: Supplemental Table S3.** The most common calls content in each call category.

**Additional file 4: Supplemental Figure S1.** Flow diagram describing the management of calls made to the ICDC call center.

## Data Availability

The data generated during the operation of the call center include identifying information on callers and are therefore not publicly available. A limited data set stripped of identifying information may be made available from the corresponding author on reasonable request.

## Abbreviations

COVID-19: Novel Coronavirus Disease 2019
HMO: Health maintenance organization
HQ: Headquarters
ICDC: Israel Center for Disease Control
MOH: Israel Ministry of Health
PPE: Personal protective equipment
SARS-CoV-2: Severe acute respiratory syndrome coronavirus 2

## References

[1] Patel A, Jernigan DB (2020). Initial public health response and interim clinical guidance for the 2019 novel coronavirus outbreak - United States, December 31, 2019-February 4, 2020. MMWR Morb Mortal Wkly Rep.

[2] World Health Organization. Coronavirus (COVID-19) situation reports. https://www.who.int/emergencies/diseases/novel-coronavirus-2019/situation-reports (Accessed 16 June 2020).

[3] Cucinotta D, Vanelli M (2020). WHO declares COVID-19 a pandemic. Acta Bio Med.

[4] Revere (2011). Public health emergency preparedness and response communications with health care providers: a literature review. BMC Public Health.

[5] Israel Ministry of Health 2007. Health system preparedness plan for pandemic influenza [Hebrew]. https://www.health.gov.il/Subjects/emergency/preparation/DocLib/tora/BIO_TORA_PANDEMIC_FLU.pdf?fbclid=IwAR3r2CjVVEkaItTXuVrUNMS5rbQOFKzzVcmaZlS07TpyxYJLdX_RjqckFVY%20 (Accessed 12 July 2020).

[6] Israel Government Databases. COVID-19 Database https://data.gov.il/dataset/covid-19. Accessed 26 Oct 2020.

[7] National Insurance Institute. Distribution of National Health Insurance Funds, 05.01.2020 [Hebrew]. https://www.btl.gov.il/Mediniyut/Situation/haveruth1/2020/Pages/default.aspx. Accessed 15 July 2020.

[8] (Israel Ministry of Health. Kol HaBriut: Telephone service center. https://www.health.gov.il/PniyotHazibur/Pages/CallCenter.aspx [Hebrew] Last accessed 21 September 2020).

[9] (Israel Ministry of Health. Guidelines, directives and information for medical personnel. https://govextra.gov.il/ministry-of-health/corona/corona-virus/medical-guidelines-corona/).

[10] Clalit Health Services. The Coronavirus Outbreak: important telephone numbers [Hebrew]. https://www.clalit.co.il/he/info/Pages/corona_contact.aspx (Accessed 16 June 2020).

[11] Macabbi Healthcare Services. Coronavirus- Guidelines for coping [Hebrew]. https://www.maccabi4u.co.il/38785-he/Maccabi.aspx (Accessed 16 June 2020).

[12] Meuhedet Healthcare services. Coronavirus: Updates, information, frequently asked questions [Hebrew]. https://www.meuhedet.co.il/%D7%94%D7%9E%D7%92%D7%96%D7%99%D7%9F/%D7%A0%D7%92%D7%99%D7%A3-%D7%A7%D7%95%D7%A8%D7%95%D7%A0%D7%94/ (Accessed 16 June 2020).

[13] Leumit Healthcare Services. Coronavirus. [Hebrew] https://www.leumit.co.il/heb/Life/FamilyHealth/familyhealth/coronavirus/ (Accessed 16 June 2020).

[14] Zimmermann P, Curtis N (2020). COVID-19 in children, pregnancy and neonates: a review of epidemiologic and clinical features. Pediatr Infect Dis J.

[15] Itelman E, Wasserstrum Y, Segev A, Avaky C, Negru L, Cohen D (2020). Clinical characterization of 162 COVID-19 patients in Israel: preliminary report from a large tertiary center. Isr Med Assoc J.

[16] Israel Ministry of Health. Family Medicine Physicians 2018. https://www.health.gov.il/PublicationsFiles/FamilyHealth_2018.pdf (Accessed 16 June 2020).

[17] Kain NA, Jardine CG (2020). “Keep it short and sweet.” Improving risk communication to family physicians during public health crises. Can Fam Physician.

[18] Baseman J (2016). A randomized controlled trial of the effectiveness of traditional and mobile public health communications with health care providers. Disaster Med Public Health Preparedness.

[19] Baseman (2013). Public health communications and alert fatigue. BMC Health Serv Res.

[20] Al-Abri SS, Kurup PJ, Al Manji A, Al Kindi H, Al Wahaibi A, Al Jardani A (2020). Control of the 2018-2019 dengue fever outbreak in Oman: a country previously without local transmission. Int J Infect Dis.

[21] Lau LL, Hung N, Go DJ, Ferma J, Choi M, Dodd W (2020). Knowledge, attitudes and practices of COVID-19 among income-poor households in the Philippines: a cross-sectional study. J Glob Health.

[22] Walter D, Böhmer MM, Reiter S, Krause G, Wichmann O. Risk perception and information-seeking behaviour during the 2009/10 influenza A(H1N1)pdm09 pandemic in Germany. Euro Surveill. 2012;17(13):20131. 22490383

[23] Fatiregun AA, Olowookere SA, Oyebade AO (2011). Pandemic influenza a (H1N1): knowledge among senior health workers at a secondary health care institution in southwest, Nigeria. Afr Health Sci.

[24] World Health Organization. COVID-19 Health system response monitor: What channels are countries using to communicate with the public and at what frequency? https://analysis.covid19healthsystem.org/index.php/2020/07/03/what-channels-are-countries-using-to-communicate-with-the-public-and-at-what-frequency/. [Last accessed 08 July 2020].

[25] US Centers for Disease Control and Prevention. CDC clinical call center. https://www.cdc.gov/coronavirus/2019-ncov/downloads/hcp/Clinician-On-Call-Center-508.pdf [last accessed 08 July 2020].

[26] British Association of Dental Nurses. https://www.badn.org.uk/News/COVID-19/NHS-helpline-for-healthcare-staff.aspx (last accessed 14 July 2020).

[27] Seidl IA (2010). A strategy for real time improvement (RTI) in communication during the H1N1 emergency response. Aust Health Rev.

