# Supplementary material for: A COVID-19 call center for healthcare providers: dealing with rapidly evolving health policy guidelines
Source: Isr J Health Policy Res. 2020 Dec 2;9:73. doi: 10.1186/s13584-020-00433-x (PMC7709808; doi:10.1186/s13584-020-00433-x)
Supplement: Supplementary file 1 — Additional file 1: Supplemental Table S1. Timeline of events and guidelines related to the national response to COVID-19. [file 13584_2020_433_MOESM1_ESM.docx]

Table 1S

| **Date** | **Events/MOH recommendations/directives for the general public** | **MOH directives for the health care system** |
| --- | --- | --- |
| 23-Jan-20 |  | COVID-19 case definition and first set of MOH medical and laboratory guidelines are distributed |
| 24-Jan-20 | The MOH bans travel to Wuhan, China, and recommends against unnecessary travel to other parts of China |  |
| 27-Jan-20 | COVID 19 added to the list of notifiable infectious diseases |  |
| 30-Jan-20 | WHO declares the novel coronavirus outbreak to be a Public Health Emergency of International Concern on 30 January 2020 (2 ) |  |
| 02-Feb-20 | Travelers returning from China are required to remain in home quarantine for 14 days |  |
| 03-Feb-20 |  | Suspected case requiring PCR test and isolation defined as symptomatic individual who returned from China or had been in contact with confirmed case within 14 days prior to symptom onset |
|  |  | Suspected cases referred to hospital ED for PCR test |
| 04-Feb-20 | First quarantine unit for COVID-19 patients is opened at Sheba Medical Center |  |
| 05-Feb-20 | ICDC call center for healthcare professionals is operational |  |
| 07-Feb-20 |  | Definition of suspected case requiring PCR test and isolation expanded to include symptomatic persons returning from Hong-Kong, Macau, Japan, Taiwan, Thailand, Singapore and South Korea within 14 days prior to symptom onset |
| 16-Feb-20 | Home quarantine requirement expanded to include travelers returning from Thailand, Hong Kong, Singapore and Macau |  |
|  | Home quarantine requirement expanded to include persons known to have been in contact with a confirmed COVID-19 |  |
| 20-Feb-20 | 11 Israeli citizens returned from the Diamond Princess cruise ship and placed in isolation at Sheba Medical Center; one of the passengers identified as Israeli COVID-19 case #1 |  |
| 23-Feb-20 |  | Definition of suspected case requiring PCR test and isolation expanded to include symptomatic persons returning from Italy and Australia |
|  |  | Magen David Adom emergency services (MDA) assumes the responsibility for in-home sample collection for PCR testing from suspected cases |
| 27-Feb-20 | First case of an Israeli citizen returning from Italy diagnosed with COVID-19. |  |
|  | Home quarantine requirement expanded to include persons returning from Italy |  |
|  | First locally-acquired COVID 19 case diagnosed (documented exposure to a returnee from Italy) |  |
| 01-Mar-20 |  | MDA establishes COVID-19 hotline for PCR testing-suspected cases instructed to contact MDA for testing rather than visiting physician offices or ED |
| 04-Mar-20 | Home quarantine requirement expanded to include persons returning from Austria, Spain, France, Germany and Switzerland |  |
|  | Overseas travel ban imposed on healthcare workers and government employees |  |
|  | Events and gatherings of more than 5000 individuals and international conferences in Israel prohibited |  |
| 05-Mar-20 |  | Definition of suspected case requiring PCR test and isolation expanded to include 1) symptomatic individuals returning from Austria, Spain, France, Germany and Switzerland, 2) Patients hospitalized with severe respiratory illnesses without known exposures |
| 09-Mar-20 | Home quarantine requirement expanded to include all persons returning from outside Israel |  |
|  | Entry to Israel for foreign nationals limited to those with a proven ability to remain in quarantine for 14 days |  |
| 10-11 Mar-2020 | Purim holiday |  |
| 11-Mar-20 | WHO declares COVID-19 outbreak a global pandemic (3 ) |  |
|  | Events and gathering are limited to 100 people |  |
|  | Sports events are to take place without audience |  |
| 12-Mar-20 | All persons required to wear a mask in public | PCR testing criteria expanded to include symptomatic persons returning from overseas, symptomatic persons treated clinics participating in MOH sentinel surveillance program |
|  |  | Healthcare workers (HCWs) in outpatients settings directed to wear surgical masks (not N95 masks) when evaluating and treating suspected cases |
| 15-Mar-20 | Shopping centers, restaurants, educational institutions closed. Pharmacies, supermarkets and grocery stores remain open. |  |
|  | Public gatherings restricted to 10 people |  |
| 17-Mar-20 |  | Individuals with fever (38 deg. C or above) and respiratory symptoms in the absence of foreign travel or contact with a COVID-19 confirmed case required to remain at home until 48 hours after resolution of the fever |
|  |  | Individuals and providers advised to minimize visits to physician offices and use telemedicine services when possible |
|  |  | Services such as physical therapy, occupational therapy or child development to be provided only in urgent situations, treatment in groups suspended |
| 25-Mar-20 |  | Criteria for PCR testing expanded to include symptomatic health care workers regardless of exposure status |
|  |  | Criteria for recovery from COVID-19 are defined |
|  |  | Individuals aged 70 years and older advised to remain at home |
|  |  | Guidelines for home hospitalization of COVID-19 confirmed patients are distributed |
| 01-Apr-20 |  | Criteria for PCR testing expanded to include employees in long-term care facilities and residents of long-term care facilities in which cases have been diagnosed |
|  |  | MOH guidelines specify limited situations in which inhalation therapy should be provided and the required protective equipment to be used in these situations |
| 08-09 Apr-2020 | Start of the Passover holiday; nationwide lockdown | Criteria for PCR testing expanded to include persons transferred to long-term care facilities from hospitals or the community |
|  |  | Modified MOH guidelines require all health care workers to wear a surgical masks during patient care activities |
| 14-15 Apr-2020 | End of the Passover holiday; nationwide lockdown |  |
|  |  | Criteria for PCR testing expanded to include persons living in geographic areas with high COVID-19 incidence or known clusters, asymptomatic persons living or working in closed facilities with known COVID-19 incidence, and symptomatic persons living or working in closed facilities. |
| 24-Apr-20 | Start of the month of Ramadan |  |
|  | Limited re-opening of non-essential businesses |  |
| 27-Apr-20 |  | Expansion of medical and nursing services provided in the community, with preference giving to telemedicine, in particular for at-risk populations |
| 28-Apr-20 | Israel Memorial Day; participation in ceremonies at military cemeteries curtailed |  |
| 29-Apr-20 | Israel Independence day; nationwide lockdown |  |
| 04-May-20 | Opening of additional non-essential businesses and activities |  |
|  | Opening of kindergartens and elementary schools 1^st^-3^rd^ grade |  |
