# Supplementary material for: A COVID-19 call center for healthcare providers: dealing with rapidly evolving health policy guidelines
Source: Isr J Health Policy Res. 2020 Dec 2;9:73. doi: 10.1186/s13584-020-00433-x (PMC7709808; doi:10.1186/s13584-020-00433-x)
Supplement: Supplementary file 2 — Additional file 2: Supplemental Table S2. Categories of questions raised by callers to the ICDC call center (N=6,933). [file 13584_2020_433_MOESM2_ESM.docx]

**Table 2S. Categories of questions raised by callers to the ICDC call center (N=6,933)**

| **Question category** | **N** | **%** |
| --- | --- | --- |
| Guidance on implementing MOH guidelines | 1727 | 24.9 |
| Guidelines for quarantine | 1494 | 21.6 |
| Definition of a suspected case | 1426 | 20.6 |
| SARS-Cov2 testing | 979 | 14.1 |
| Locating PCR results | 457 | 6.6 |
| Managing suspected or confirmed cases in the community | 371 | 5.4 |
| Management of persons exposed to a known case | 162 | 2.3 |
| Criteria for recovered case | 93 | 1.3 |
| Use of personal protective equipment | 81 | 1.2 |
| Other | 143 | 2.1 |
| Total | 6933 | 100.0 |
