# Supplementary material for: A COVID-19 call center for healthcare providers: dealing with rapidly evolving health policy guidelines
Source: Isr J Health Policy Res. 2020 Dec 2;9:73. doi: 10.1186/s13584-020-00433-x (PMC7709808; doi:10.1186/s13584-020-00433-x)
Supplement: Supplementary file 3 — Additional file 3: Supplemental Table S3. The most common calls content in each call category. [file 13584_2020_433_MOESM3_ESM.docx]

**Table 3S. The most common calls content in each call category**

| **Guidelines for home quarantine** |
| --- |
| What are the guidelines for home quarantine? |
| Travelers returning from country "X", is home quarantine required? |
| Are there any restrictions on household members of an individual in home quarantine? |
| Are there any restrictions on household members of an individual in home quarantine, if the household members are aged 60 and above/ with chronic diseases/ suppressed immune system? |
| Does close contact with a healthy person, who is now in home quarantine, require home quarantine? |
| Should individuals with fever and/or respiratory symptoms after a close contact with other symptomatic or asymptomatic individuals who are now in home quarantine, be required to enter home quarantine themselves? |
| Should a close contact with a healthy person who had a contact with a COVID-19 patient, be required to enter home quarantine? |
| What is a close contact? |
| Under what circumstances should contact of a COVID-19 patient be required to enter home quarantine? (Having been within a distance of 2 meters for few minutes / Having been in the same space with a person diagnosed with COVID-19 for at least 15 minutes) |
| What is the duration (date to date) of Quarantine for an individual returning from a foreign country? |
| What should symptomatic individuals who returned from abroad and completed 14 days of quarantine do? |
| What should symptomatic travelers returning from countries which are not on the current list that requires them to be in home quarantine, but had a connecting flight through countries which are on that list, do? Should they be required to enter home quarantine? |
| How can travelers returning from countries on the home quarantine list and commit to not using public transportation, get home if they do not have a car? |
| What should individuals in home quarantine, who require a non-COVID-19 related medical attention such as dialysis, labor/delivery, or emergency management, do? |
| Should close contact with a SARS-CoV2-positive individual require home quarantine and for what duration (date to date)? |
| Should individuals with a fever and/or respiratory symptoms who returned from a foreign country and tested negative for SARS-CoV2, be required to remain in home quarantine for the entire 14 days period? |
| Home quarantine violation questions. |
| Should Individuals who returning from foreign countries not included in the home quarantine list and developed fever and/or respiratory symptoms during the following week, start home quarantine? |
| Individuals who received a text (SMS) or voice message informing them of exposure to a COVID-19 patient and instructing them to remain in home quarantine for 14 days, think this is a mistake. What should/can be done? |
| I have received a message (SMS) that I have to enter isolation, but I am a medical professional, and on the date/time specified by the message I was fully protected by PPE. What should I do? |
| What should individuals with fever and/or respiratory symptoms who do not know whether they have been in contact with a COVID-19 patient and have not returned from a foreign country, do? |
| Who will compensate individuals in home quarantine for missed work days? |
| Can a physician examine a quarantined patient in the patient's home? |
| **Definition of a suspected case** |
| Do individuals with fever and/or respiratory symptoms following a contact with symptomatic individuals who were required to remain in home quarantine for 14 days following a foreign travel, meet the case definition? |
| Does a close contact of an individual or a group that tested positive SARS-CoV2, meet the case definition? |
| Do individuals with fever and/or respiratory symptoms after a contact with other asymptomatic individuals returning from foreign countries and were required to remain in home quarantine, fulfill the case definition? |
| Do individuals with fever and/or respiratory symptoms who had a contact with other asymptomatic individuals who were home quarantined due to a contact with a confirmed COVID19 case, fulfill the case definition? |
| Do Individuals with fever and/or respiratory symptoms after a contact with other symptomatic or asymptomatic individuals who themselves were in contact with individuals returning from foreign travel and were required to remain in home quarantine, fulfill the case definition? |
| Do Individuals with fever and/or respiratory symptoms after a contact with other symptomatic or asymptomatic individuals who themselves were in contact with other individuals who were home quarantined following contact with a confirmed COVID19 case, fulfill the case definition? |
| Do individuals with fever /or respiratory symptoms following a contact with other asymptomatic individuals who returned from foreign travel and completed home quarantine, fulfill the case definition? |
| Do individuals returning from foreign countries which are not included in the home quarantine list and developed fever and/or respiratory symptoms in the following week, fulfill the case definition? |
| Do individuals returning from foreign countries which are included in the home quarantine list and developed fever and/or respiratory symptoms in the following days/week, fulfill the case definition? |
| Do asymptomatic individuals who returned from foreign countries, before these countries were included in the list of required home quarantine, meet the case definition? |
| Do individuals diagnosed with pneumonia after contact with asymptomatic individuals who returned from foreign travel and were required to remain in home quarantine, fulfill the case definition? |
| Do individuals who had a contact with individuals who were isolated due to close contact with a confirmed COVID19 case, fulfill the case definition? |
| Calls regarding patients with pneumonia who do not fulfill case definition. |
| Calls regarding patients with chest pain and dyspnea who do not fulfill case definition. |
| Individuals with fever and /or respiratory symptoms, who do not fulfill case definition. |
| Do individuals with fever and/ respiratory symptoms, without an epidemiological connection, but who work as airport employees, service providers or taxi/bus drivers, meet the case definition? |
| Do individuals with fever and/ respiratory symptoms and less than 15 minutes contact with a confirmed COVID-19 patient, meet the case definition? |
| Calls regarding healthcare workers with clinical symptoms with or without case definition. |
| Do symptomatic travelers returning from countries which do not require them to be in home quarantine, but transited through countries that require quarantine, fulfill the case definition? |
| **Exposure of a confirmed case** |
| Requesting information, including exact path of confirmed COVID-19 cases (for the purpose of entering home quarantine) |
| Reporting exposure to a confirmed COVID-19 patient and requesting instructions |
| Calls from nursing homes and assisted living facilities requesting guidelines regarding residents returning from hospitals were they were exposed to confirmed COVID-19 patients. |
| Inquiry of the guidelines for physicians who were exposed to confirmed COVID-19 patients, before the patients were tested. |
| Inquiry of the guidelines for managing individuals who developed symptoms following exposure to confirmed COVID-19 patients. |
| What are the criteria for SARS-CoV2 testing following exposure to a confirmed COVID-19 patient? |
| Guidelines for household members of a confirmed COVID-19 patient |
| Guidelines for managing patients treated by medical personnel who were found to be SARS-CoV2-positive |
| Guidelines for managing symptomatic patients who were in contact with their medical provider who was found to be SARS-CoV2-positive |
| Receiving text/voice massages regarding exposure to a confirmed COVID-19 patient without location |
| Receiving false text/voice massages regarding exposure to a confirmed COVID-19 patient |
| Individuals receiving text/voice massages regarding exposure to a confirmed COVID-19 patient in clinics/pharmacies, without the clinics/pharmacies receiving similar massages. |
| Conflicting guidelines regarding exposure to confirmed COVID-19 patients |
| **Personal protective equipment (PPE)** |
| Guidelines for effective use of PPE |
| Effectiveness of different kinds of masks and gowns |
| Requests for upgrading the PPE requirements for medical personnel in MOH guidelines |
| Requesting information regarding the risk to contact SARS-CoV2 using the PPE specified in MOH Guidelines |
| Lack of basic PPE in Hospitals and community clinics |
| Lack of advanced PPE in Hospitals and community clinics |
| PPE for travel to foreign countries |
| Repeat use of PPE |
| Request for guidelines for PPE use by workers providing non-medical service to the public (airport, pharmacy, transportation etc.) |
| Where can PPE be privately purchased? |
| **SARS CoV2 testing** |
| Guidelines for SARS-CoV2 testing |
| Asking about SARS-CoV2 testing because of relevant symptoms alone |
| Asking about SARS-CoV2 testing because of relevant symptoms couples with visiting public places |
| Asking about SARS-CoV2 testing following contact with a contact of a COVID-19 patient |
| Requests for SARS-CoV2 testing in the community |
| Requests for repeat SARS-CoV2 testing due to loss or technical difficulty of a previous test |
| **SARS CoV2 test results** |
| How do patients receive SARS-CoV2 test results? |
| How can a physician receive the results of SARS-CoV2 test results of his patient that was not performed by the patient's HMO? |
| How long does it take to receive SARS-CoV2 test results? |
| Requesting guidelines regarding loss of SARS-CoV2 tests |
| **Managing confirmed/suspected cases** |
| Managing individuals who returned from foreign countries that are not on the list of home quarantine and arrive to outpatient clinics with fever/respiratory symptoms |
| Managing a patient who fulfills the criteria of a suspected case who arrived at an HMO outpatient clinic |
| Disinfecting an HMO outpatient clinic after the visit of a patient suspected to have COVID-19 |
| Managing patients who disobey quarantine in HMO outpatient clinics |
| Guidelines for examining a patient whose family member has COVID-19 |
| Managing individuals who tested negative for SARS-CoV2 and arrive to the clinic with fever/respiratory symptoms |
| Concerns of medical, laboratory, imaging, pharmacy and dental care personnel, from exposure to COVID-19 suspected cases |
| Performing routine medical procedures at outpatient clinics for patients who are in home quarantine following a foreign travel. |
| Managing patients with fever/respiratory symptoms who have had contact with a quarantined individual |
| The process of sending a patient suspected to have COVID-19 to the emergency department |
| Remote care of quarantined patients with fever/respiratory symptoms with/without epidemiological circumstances (foreign travel or close contact with a COVID-19 patient) |
| Requests for sick notes/ medical permissions to return to work. |
| Referrals of patients with fever/respiratory symptoms to clinical/laboratory/imaging services |
| **Implementing MOH guidelines** |
| Requests for guidelines for foreign travel |
| Requests for guidelines for individuals returning from foreign countries including countries of connection flights |
| Requests for guidelines for managing patients with respiratory symptoms without epidemiological circumstances |
| Requests for guidelines for 'Mikve' ritual immersion |
| Requests for work guidelines for medical teams in clinics and urgent care services |
| Request for guidelines for patients returning from foreign countries |
| Definition of close contact |
| Guidelines for use of global statement of illness |
| Guidelines for action after exposure to COVID-19 cases in a football stadium in Israel and abroad |
| Guidelines for transportation of individuals requiring home quarantine. |
| Discrepancy between the written guidelines and the version advertised in the news and the websites |
| Clarification of guidelines |
| Request for guidelines for private and public swimming pools |
| Guidelines forbidding foreign travel of healthcare workers including reimbursement of expenses due to travel cancellation |
| Request for guidelines for healthcare workers who are at risk due to age or background diseases. |
| Request for guidelines for communal dining rooms |
| Individuals requiring quarantine due to guidelines update |
| Request for guidelines for operating educational facilities/institutions |
| Request for guidelines for visiting nursing homes |
| Request for guidelines for repairs of examination rooms (window, air conditioner etc) |
| Request for guidelines for work and PPE at dental clinics |
| Request for guidelines for a household with one family member in home quarantine |
| Request for guidelines for visiting hospitalized patients |
| Request for guidelines for ambulatory care |
| Request for guidelines for treating foreign patients |
| Request for guidelines for laundry of individuals in home quarantine |
| Request for guidelines regarding gatherings (weddings, funerals, parties and conferences) |
| Request for guidelines regarding individuals returning from conferences in foreign countries |
| Request for guidelines for special populations (dialysis patients, oncology patients, pregnant women) |
| Request for guidelines for patients with fever/respiratory symptoms without epidemiological circumstances |
| Request for guidelines for tourists that are not Israeli citizens |
| Request for guidelines for operation of operating rooms |
| Request for guidelines for handling mail/packages/merchandise originating in foreign countries |
| Request for guidelines for using public transportation |
| Request for guidelines for preparing and usage of disinfectants |
| Request for guidelines regarding public service workers (municipalities, electric companies, post office, banks etc.) |
| Request for guidelines regarding contact with an individual who was in contact with a COVID-19 patient |
| Request for guidelines regarding afterschool and sports activities |
| Request for guidelines for operating senior citizens and rehabilitation day centers |
| Request for guidelines for operating central air conditioning systems |
| Request for guidelines for managing patients not fulfilling the case definition of a suspected case in outpatient clinics |
| Request for guidelines regarding exemption from quarantine for individuals coming from foreign countries due to death of a first degree relative |
| Request for guidelines for screening patients about foreign travel and/or respiratory symptoms prior to their clinic visit |
| Request for guidelines/concern regarding exposure to individual s from South-East Asia |
| Request for information regarding SARS-CoV2 transmission mode and likelihood of transmission from symptomatic and asymptomatic SARS-CoV2-positive individuals |
| Request for information regarding medications for the treatment of COVID-19 patients |
| Request for information regarding medications that are contraindicated in COVID-19 patients |
| **Implementing MOH guidelines** |
| Requests for guidelines for foreign travel |
| Requests for guidelines for individuals returning from foreign countries including countries of connection flights |
| Requests for guidelines for managing patients with respiratory symptoms without epidemiological circumstances |
| Requests for guidelines for 'Mikve' ritual immersion |
| Requests for work guidelines for medical teams in clinics and urgent care services |
| Request for guidelines for patients returning from foreign countries |
| Definition of close contact |
| Guidelines for use of global statement of illness |
| Guidelines for action after exposure to COVID-19 cases in a football stadium in Israel and abroad |
| Guidelines for transportation of individuals requiring home quarantine. |
| Discrepancy between the written guidelines and the version advertised in the news and the websites |
| Clarification of guidelines |
| Request for guidelines for private and public swimming pools |
| Guidelines forbidding foreign travel of healthcare workers including reimbursement of expenses due to travel cancellation |
| Request for guidelines for healthcare workers who are at risk due to age or background diseases. |
| Request for guidelines for communal dining rooms |
| Individuals requiring quarantine due to guidelines update |
| Request for guidelines for operating educational facilities/institutions |
| Request for guidelines for visiting nursing homes |
| Request for guidelines for repairs of examination rooms (window, air conditioner etc) |
| Request for guidelines for work and PPE at dental clinics |
| Request for guidelines for a household with one family member in home quarantine |
| Request for guidelines for visiting hospitalized patients |
| Request for guidelines for ambulatory care |
| Request for guidelines for treating foreign patients |
| Request for guidelines for laundry of individuals in home quarantine |
| Request for guidelines regarding gatherings (weddings, funerals, parties and conferences) |
| Request for guidelines regarding individuals returning from conferences in foreign countries |
| Request for guidelines for special populations (dialysis patients, oncology patients, pregnant women) |
| Request for guidelines for patients with fever/respiratory symptoms without epidemiological circumstances |
| Request for guidelines for tourists that are not Israeli citizens |
| Request for guidelines for operation of operating rooms |
| Request for guidelines for handling mail/packages/merchandise originating in foreign countries |
| Request for guidelines for using public transportation |
| Request for guidelines for preparing and usage of disinfectants |
| Request for guidelines regarding public service workers (municipalities, electric companies, post office, banks etc.) |
| Request for guidelines regarding contact with an individual who was in contact with a COVID-19 patient |
| Request for guidelines regarding afterschool and sports activities |
| Request for guidelines for operating senior citizens and rehabilitation day centers |
| Request for guidelines for operating central air conditioning systems |
| Request for guidelines for managing patients not fulfilling the case definition of a suspected case in outpatient clinics |
| Request for guidelines regarding exemption from quarantine for individuals coming from foreign countries due to death of a first degree relative |
| Request for guidelines for screening patients about foreign travel and/or respiratory symptoms prior to their clinic visit |
| Request for guidelines/concern regarding exposure to individual s from South-East Asia |
| Request for information regarding SARS-CoV2 transmission mode and likelihood of transmission from symptomatic and asymptomatic SARS-CoV2-positive individuals |
| Request for information regarding medications for the treatment of COVID-19 patients |
| Request for information regarding medications that are contraindicated in COVID-19 patients |
| **Definition of a recovered COVID-19 case** |
| How is a recovered COVID-19 case defined |
| How may SARS-CoV2 tests should be performed and at which intervals in order to determine recovery from COVID-19 |
| How to manage exposure to a Covid-19 case after recovering from COVID-19? |
| Who is in charge of performing SARS-CoV2 testing for patients recovering from COVID-19? |
| Returning to routine life after recovery from COVID-19 |
| **Other** |
| Callers of the general public wo were referred to the hotline dedicated to the general public (*5400) |
| Callers verifying that the call center is operational |
| Calls that were unrelated to COVID-19 |
