# Supplementary figures and images for: A COVID-19 call center for healthcare providers: dealing with rapidly evolving health policy guidelines
Source: Isr J Health Policy Res. 2020 Dec 2;9:73. doi: 10.1186/s13584-020-00433-x (PMC7709808; doi:10.1186/s13584-020-00433-x)

## Slide 1
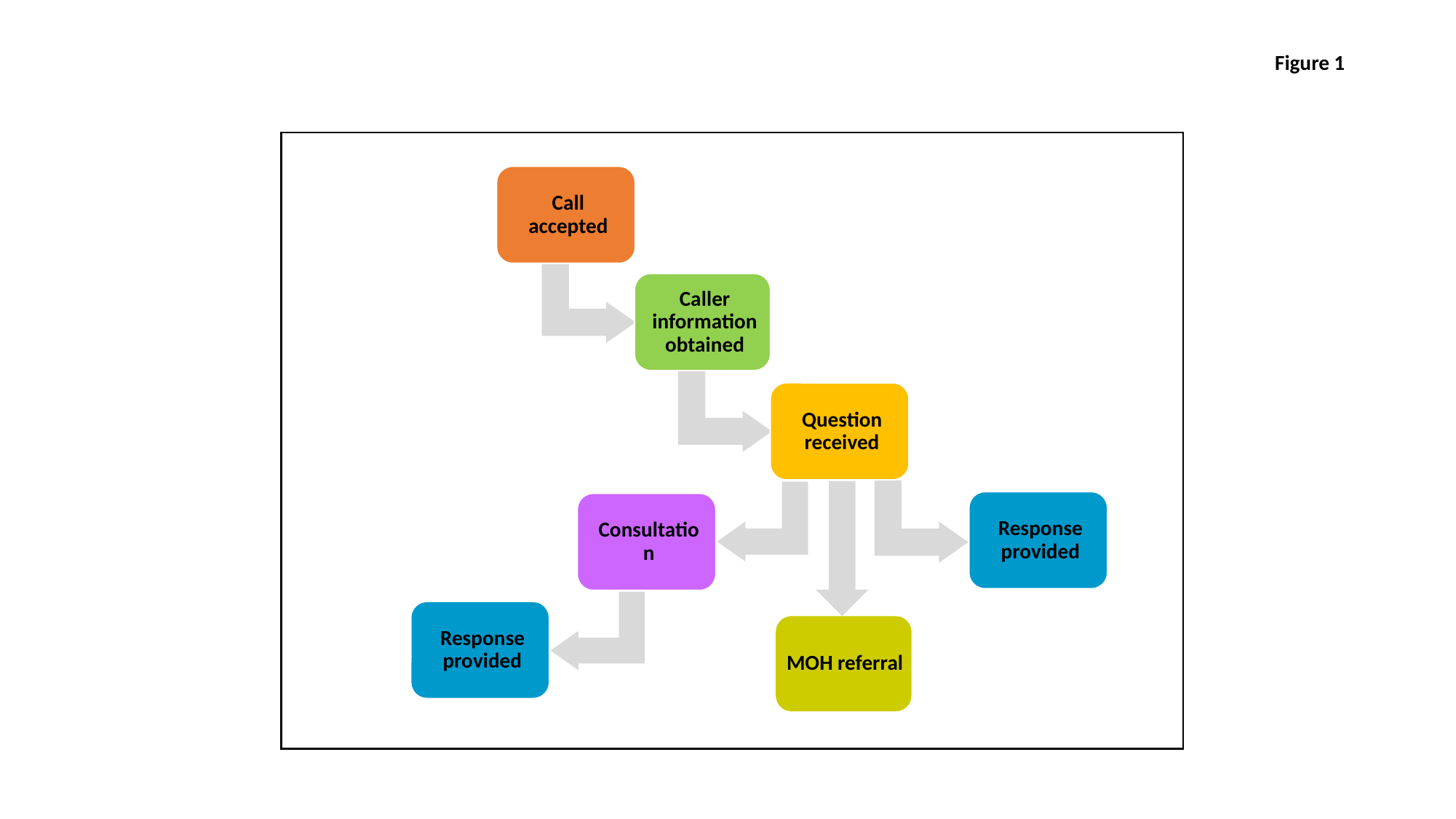

Figure 1
MOH referral

Supplement: Supplementary file 4 — Additional file 4: Supplemental Figure S1. Flow diagram describing the management of calls made to the ICDC call center. [file 13584_2020_433_MOESM4_ESM.pptx]
